# Decision criteria for the selection of analytical instruments used in clinical chemistry: V The interaction of new instrumentation with laboratory infra-structure: modelling and simulation for planning of laboratory functions

**DOI:** 10.1155/S1463924680000119

**Published:** 1980

**Authors:** Bengt Sandblad

**Affiliations:** Dept of Automatic Control and Systems Analysis Institute of Technology Uppsala University Box 534 Uppsala S- 751 Sweden


### Decision criteria for the selection of analytical instruments used in clinical chemistry

### V The interaction of new instrumentation with laboratory infra-structure : modelling and simulation for planning of laboratory functions

**Bengt Sandblad**
*Dept of Automatic Control and Systems Analysis, Institute of Technology, Uppsala University, Box 534, S-751 Uppsala, Sweden*

CLINICAL chemistry laboratories are facing increasing workloads in addition to demands for higher quality, clinical evaluation and shorter request-to-report times. The cost of health care is also increasing and it is important that advanced methods for planning and optimizing laboratory activities, as well as cost/benefit analysis, must be used in the future.

The basis for all analyses of the problems involved and for the development of methods for planning activities, is a formalized description, a *model*, of the total laboratory function. Such models provide a language in which it is possible to describe the system and its function, and enable a discussion in relevant terms.

A model of the clinical laboratory system (or of any other medical service system), can also be used for test purposes in the hospital environment, i.e. computer based *simulation experiments*. Such experiments, during which the complex and highly dynamic processes of the modelled functions are analysed, can significantly improve the knowledge of the functions of the system under study, and therefore enhance the planning process.

These studies, aimed at planning, scheduling, optimizing, etc., the laboratory functions, can be performed at different levels. In Figure 1, two such levels are pointed out, corresponding to two different levels of criteria. The *internal effectiveness* corresponds to studies of the internal laboratory functions. The *external effectiveness* corresponds to studies concerning the coordination and synchronization of different service functions to the needs and expectations of the doctor, the patient and the patient management process.

In this paper a method for studying internal effectiveness will be presented and discussed in relation to an example (p.11).

### Method

Formal models of the laboratory system under consideration are the basis for all simulation experiments. They can be complex and difficult to handle but the use of the more intelligent programming languages, which include powerful simulation facilities, greatly assists programming. SIMULA is an example of such a language and has been used in the studies presented here.

The simulation programs can be used for computer based experiments of different proposed laboratory functions. To obtain statistically acceptable results to the actual situation under consideration, it is very important that the simulation experiments are well planned, and that enough attention is paid to the statistical aspects.

The results from simulation experiments must always be carefully evaluated with respect to restrictions made in the formulation of the models and to the sensitivity of the results to variations in input-data before being used in decision-making.

### Models

The models of the clinical laboratory system used for the applications described here have been previously described, [1,2], but in order to give a background for the experiments presented below, a short description of the main characteristics will be given.

For a low level of differentiation the models consist of a number of *request stations*, *transport channels* and the *operative station* (the laboratory). At the request stations, i.e. the different wards, out-patient departments etc., the requests for laboratory investigations are generated. Each request can contain a combination of different tests, i.e. a *profile* of tests.

The transport channel is used to deliver the requests to the laboratory and the results from the investigation back to the request station (Figure 2).

For studies of the internal effectiveness no further differentiation of the request station module is made. The internal structure of the operative station thus consists of specimen receiving, preparation and distribution units, analyzing channels and units for result evaluation and report preparation.

The analyzing channels can be manual or automated and of single channel or multichannel type.

The algorithms defining the functions of the laboratory within this structure consist of one for the generation of laboratory workload and others for describing the functions of the different resources inside each laboratory unit.

The generation of the laboratory workload must be such that it reflects the workload of the laboratory studied in a statistically acceptable manner.

The algorithms of the different laboratory units describe the work of the operators and the function of the equipment at a level of detail relevant for the study. In Figure 3, the main structure of the model is described. The functions of the different units can be changed to describe any function in a normal laboratory system. Examples of such functions are special priority algorithms, and restrictions in the algorithm for certain equipment and batch-functions [1].

To enable a general model to be used in problem solving activities it must be adapted to local circumstances. This is done by a set of data, giving quantitative values for defined model parameters. Relevant data are however, always difficult to obtain, and one advantage in the models used is that they themselves define the data needed, and therefore guide the collection of laboratory statistics.

A model represents an abstraction of the real system and it is not possible or meaningful to try to include all aspects of the real system. Therefore restrictions must be made. These must always be evaluated with respect to relevance in the actual study. The models used in the examples discussed here describe the normal functioning of the laboratory in sufficient detail. Functions which are not relevant to the study have been given a "black-box" representation. Very few possibilities of abnormal functioning, for example staff shortages and equipment break-downs, are included, but it is possible to perform separate studies to define the effects of these problems on the normal behaviour of the laboratory.

### Experiments

The models and methods discussed above have been used for studies in connection with a number of real planning projects. Examples of such studies are centralisation-decentralisation of laboratory resources in a hospital and a health care region, staff allocation, planning of integrated or separated emergency analyzing organizations, "bottle-neck" studies to optimize resource utilization with respect to specimen and information flow, and a prospective evaluation of suggested reorganizations, e.g. for selecting new laboratory equipment.

Some of these studies have already been described [3,4] but one will be presented here as an example. The study was performed as a part of a project to evaluate possible strategies for renewing the analytical equipment of the central laboratory of a large hospital. The effects on the laboratory functions were investigated when one of four multichannel analysers was considered. The analysers (M1, M2, M3, and M4) have different characteristics, for example speed, capacity, loading function, number of different tests, flexibility and working hours. Since the analysers have varying capacities, the experiments were repeated for another method of organization, where each of the four instruments operates in parallel with another analyser (S 1) capable of carrying out a large workload of electrolyte and serum creatinine assays.

A number of performance characteristics are of importance when studying the effects of different proposed alternative methods of organization. These determine the workload and the resource utilization in the different parts of the laboratory system and are as follows:

(1) Configuration, i.e. what equipment is used in the experiment. All other types of analyses not performed on the multichannel analysers indicated are performed on single channel analysers.
(2) Number of analysing channels necessary for performing the different types of tests.
(3) Number of secondary specimens leaving the receiving area after portioning. This is determined by the requested profiles and the organisational structure of the laboratory.
(4) Number of specimen portionings which have to be performed in the receiving area.

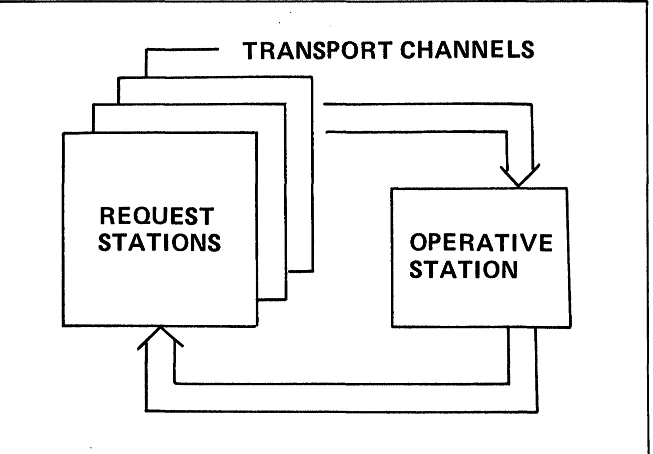

*Figure 2. The main parts of the model*

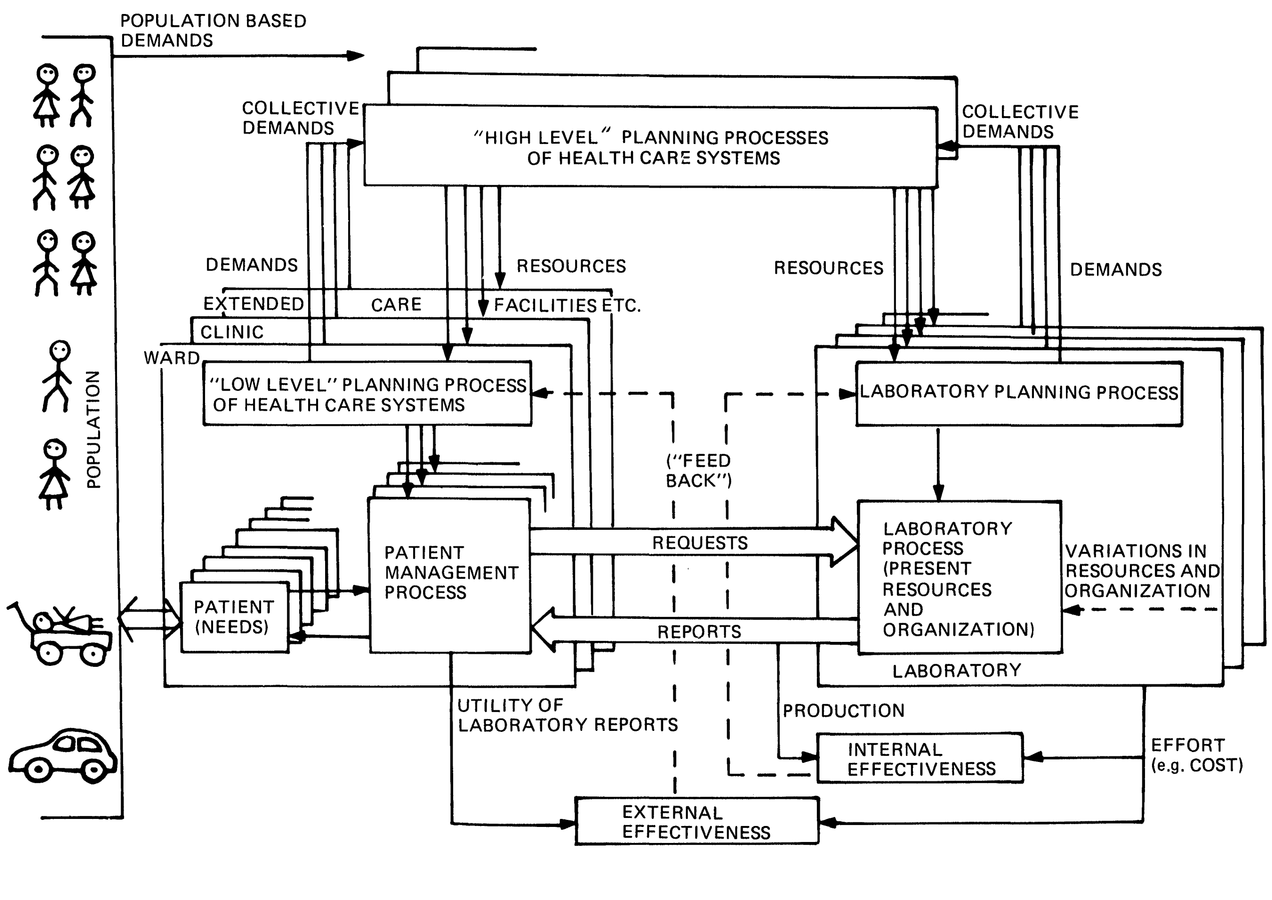

*Figure 1. Conceptual model of the health care delivery system*

(5) Performance data for the analysers themselves.
(6) Number of different types of analyses performed in the analysing channels.
(7) Number of secondary specimens transferred to the analysers.
(8) Number of requested tests.
(9) The mean number of requested tests per secondary specimen.
(10) The percentage of the total workload, measured in tests, which is performed on the analysers.

Together with the time dependent dynamic performance of the different parts of the laboratory system, these characteristics determine the total performance of the laboratory. This can then be calculated directly during the simulation experiments.

Figure 4 shows an example of some time aspects of the results. These times are functions of the workload and the structure of the laboratory, and cannot be obtained without a model describing the system as a whole. It can be seen, for example that the analysing phase of the work is finished earlier for M4 than for M2, but the reports are delivered later when M4 is used. This is because of the increased number of channels now utilizing the common resources in the report preparation area.

In another experiment, the number of requests was changed in order to simulate an increasing laboratory workload. The different proposed structures have a varying sensitivity to such changes which can be difficult to predict.

When the time periods for finishing the laboratory procedures become unacceptably long, different attempts to improve the capacity of the laboratory can be made. Experiments can be performed where the effects of variations in personnel at different locations or during peak-hours are evaluated. Different structures respond in a varying manner to such variations. The optimal set of resources in each part

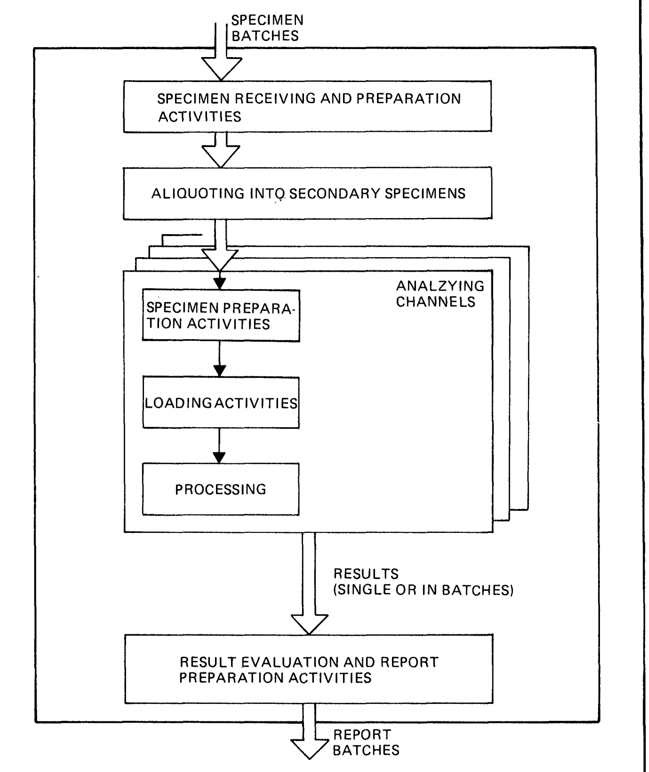

*Figure 3. Modules of the laboratory model*

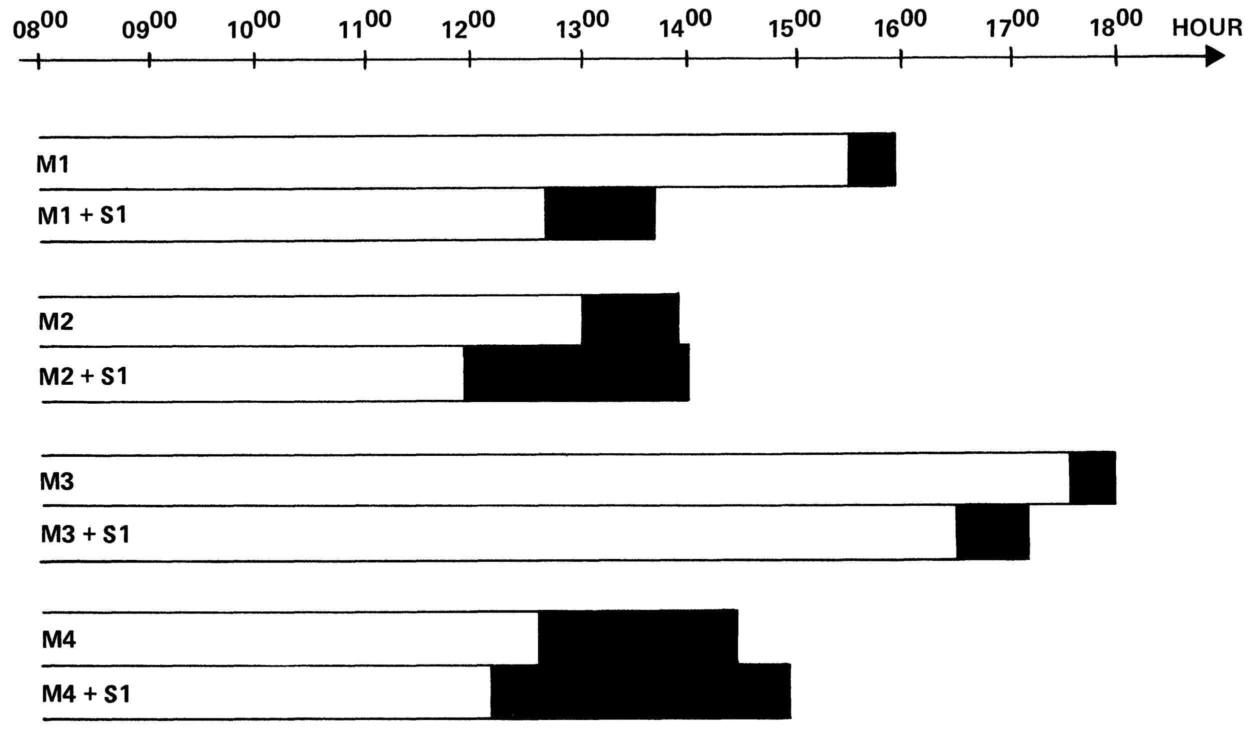

*Figure 4. Time evaluation of some proposed organizations*

of the laboratory can be determined through experiments of this kind for each proposed structure.

In order to investigate the possibility of including emergency tests in the proposed structures, some further experiments were made. It was shown that the different analysers have different capabilities under these circumstances and that some proposed structures require additional equipment for achieving an acceptable overall performance of the laboratory. This is likely to be expensive.

### Discussion

Simulation experiments are only one phase of the total evaluation project. A final decision must be based on several sets of information, some of which cannot be obtained through simulation experiments. There are several factors of importance not considered in the models used here. These include financial aspects; different reliability aspects for which special studies can be made; factors concerning the possibility of using the equipment in other circumstances than those studied, for example during the night; analytical quality aspects; and man-machine (ergonomic or environmental) aspects.

The models are applicable to most clinical laboratory systems. They can be adapted to local circumstances through the adjustment of input parameters. Results from stimulation experiments are, however, mostly specific to each studied laboratory system. For example significant variations in the request profiles can have a considerable influence on the results. The models are flexible in that they describe the laboratory system as a whole and can therefore be used for studies of different aspects of problems connected with the planning process.

Other problems can be introduced when evaluating the results from the simulation experiments. Thus a change in the layout of the specimen reception area can result in a changed reception pattern and this is particularly important when multichannel analysers are involved. Under these circumstances the conditions of evaluation are changed and the results from the simulation experiments are no longer valid. Such effects can be difficult to predict, and a sensitivity analysis of the result with respect to such variations must be made.

Another important factor not included in these studies is the evaluation with respect to the external effectiveness of the laboratory, i.e. the "medical benefit" of the report from the laboratory investigation. Some multichannel analysers produce reports in the form of test-profiles, where tests are reported which are not necessarily requested. The medical benefit of such reports is difficult to evaluate but has been extensively discussed elsewhere.

Techniques and methods for simulation studies with respect to external effectiveness are being developed [5].

REFERENCES

[1] Sandblad, B., Systems analysis and simulation of health care laboratory systems - methodology and models. UPTEC 77 13R (1977), Institute of Technology, Uppsala University. To be published in *Computer Programs in Biomedicine*.

[2] W Schneider and S Bentsson eds.: The application of computer techniques in health care. *Computer Programs in Biomedicine*, (1976) 5(3) 169-250.

[3] Sandblad, B., Modelling and simulation for planning and cost/benefit analysis of health care service systems. *Medical Informatics* (1976) 1, (1), 27-37.

[4] Sandblad, B., Hellsing, K. and de Verdier, C. H. The use of multichannel analytical equipment in clinical chemistry – a simulation study. UPTEC 77 16R (1977), Institute of Technology, Uppsala University.

[5] Bengtsson, S., Sandblad, B., Schneider, W. and Spencer, W. A. Effectiveness studies in connection with health care service functions. IFIP working conference on evaluation of efficacy of medical action. Bordeaux 14-19 May 1979.

ACKNOWLEDGEMENTS

This paper was prepared in collaboration with Uppsala University Data Centre and the Department of Clinical Chemistry, Uppsala University Hospital.

### Decision criteria for the selection of analytical instruments used in clinical chemistry

### VI Techniques for the economic evaluation of automatic analysers

**Thomas M. Craig**

*EI du Pont de Nemours & Co, Wilmington, Delaware 19898, USA*

The financial evaluation of automation is a three-step process. Firstly, it is necessary to determine present costs in the laboratory and thus provide a base with which possible alternatives can be compared. Secondly, computation of the total cost of each alternative is required, including both initial acquisition cost and operating costs. Thirdly, the cost/benefit assessments of the alternatives need be compared in the light of their ability to satisfy specific requirements.

The current laboratory costs may be considered under two headings - direct and indirect; of these, only the former is relevant to decision making on the installation of an automatic analyser. These direct costs, shown in Figure 1, include coverage of supplies, labour, reagents (including wastage), standards, controls, and any repeat or duplicate measurements required. In most cases indirect costs, such as expenditure on supervision and overheads will not change no matter which analyser is selected.

The Hospital Administrative Services Group of the American Hospital Association publishes a survey of the direct costs in hospitals. It is based on data from 1,800 hospitals. The average direct cost per test in any laboratory is determined by dividing the total direct cost by the number of tests run. As can be seen from Figure 2 (which shows the results from the last survey collecting data in the direct cost/test format which was conducted in 1976), costs varied significantly according to hospital bed size.

